# The effect of host structure on the selectivity and mechanism of supramolecular catalysis of Prins cyclizations†
†Electronic supplementary information (ESI) available: Kinetics plots are available free of charge. See DOI: 10.1039/c4sc02735c



**DOI:** 10.1039/c4sc02735c

**Published:** 2014-11-28

**Authors:** William M. Hart-Cooper, Chen Zhao, Rebecca M. Triano, Parastou Yaghoubi, Haxel Lionel Ozores, Kristen N. Burford, F. Dean Toste, Robert G. Bergman, Kenneth N. Raymond

**Affiliations:** a Chemical Sciences Division , Lawrence Berkeley National Laboratory , Department of Chemistry , University of California , Berkeley , California 94720 , USA . Email: fdtoste@berkeley.edu ; Email: rbergman@berkeley.edu ; Email: knraymond@socrates.berkeley.edu

## Abstract

The effect of host structure on the selectivity and mechanism of intramolecular Prins reactions is evaluated using K_12_Ga_4_L_6_ tetrahedral catalysts. The host structure was varied by modifying the structure of the chelating moieties and the size of the aromatic spacers. While variation in chelator substituents was generally observed to affect changes in rate but not selectivity, changing the host spacer afforded differences in efficiency and product diastereoselectivity. An extremely high number of turnovers (up to 840) was observed. Maximum rate accelerations were measured to be on the order of 10^5^, which numbers among the largest magnitudes of transition state stabilization measured with a synthetic host-catalyst. Host/guest size effects were observed to play an important role in host-mediated enantioselectivity.

## Abstract 2

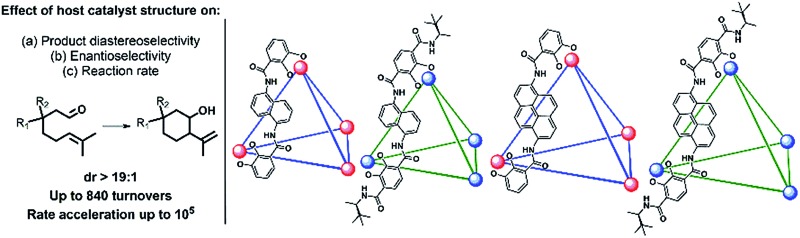
Catalyst and substrate modification, product selectivity and kinetic studies comprehensively describe a new class of terpenoid cyclase mimics.

## Introduction

Enzymes use precisely tailored binding pockets to mediate stereoselective catalysis.^1–5^ For example, terpene synthases catalyze the cyclization of simple precursors to over 70 000 known small molecule natural products.^6–8^ While these enzymes clearly demonstrate a high degree of chemical divergence, precisely how they do so is an area of continuing and fruitful investigation.

In recent years, the field of supramolecular catalysis has progressed toward understanding the role of chemical microenvironments during catalysis.^9–23^ Analogous to the active sites of many enzymes, synthetic hosts mediate catalysis through the organization of catalytically relevant functional groups that lower activation barriers relative to those that would be present in bulk solution. These strategies have relied on local concentration effects, electrostatics, p*K*_a_ shifts and the use of host–guest orientation to stabilize high-energy intermediates and transition states.

We have previously reported the use of a racemic, homochiral (Λ_4_ or Δ_4_) K_12_Ga_4_L_6_ tetrahedron ((±)-**1**) to catalyze the Prins cyclization of monoterpene derivatives.^24^ While catalytic antibodies^25–28^ have been shown to mimic key properties of terpene synthases, the tetrahedron described above acts as a purely synthetic active site mimic.^29^ In contrast to catalysis in acidic aqueous solution, which affords cyclic diol products, host-catalyzed cyclizations resulted in high selectivity for alkene products. This example of selectivity parallels that of terpene synthases such as limonene synthase.^30^ In a more recent development, a chiral ligand that self-assembles in an enantiopure fashion was prepared, affording enantiomeric terephthalamide-based hosts (Λ_4_- or Δ_4_-**2**). These hosts were observed to effect an enantioselective variant of the Prins reaction.^31^ Inhibition experiments have indicated that catalysis proceeds initially through substrate encapsulation, which is reversible and rapid.^24,32,33^ In light of these developments, investigation into the sources of the observed chemo-, diastereo-, and enantioselectivities of these reactions was pursued.

**Fig. 1:**
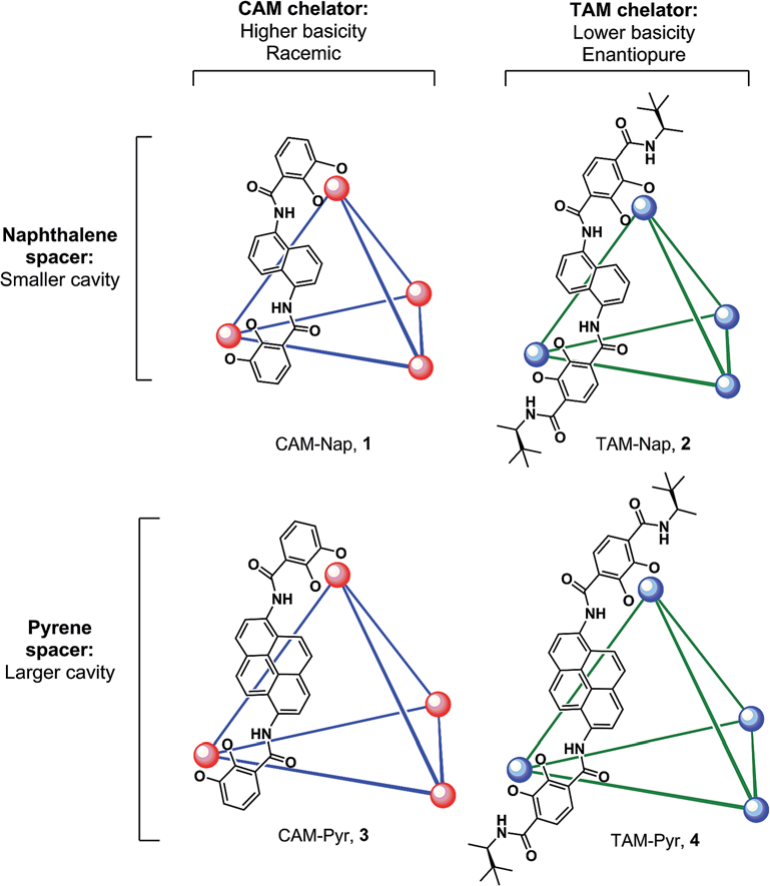
K_12_Ga_4_L_6_ assemblies discussed in this work. Spheres represent Ga^3+^ centers and lines represent ligands as depicted (CAM = catecholamide, TAM = terephthalamide, Nap = naphthalene, Pyr = pyrene). Only one ligand enantiomer is shown for **2** and **4**. Potassium ions are omitted for clarity.

We present a mechanistic study of the Prins cyclization in supramolecular host catalysts whose structures were systematically varied in the choice of chelator (CAM = catecholamide, TAM = terephthalamide) and spacer (Nap = naphthalene, Pyr = pyrene; Fig. 1). Differences in catalytic rate, as well as product chemo-, diastereo- and enantioselectivity were found. The nature of host-mediated enantioinduction was investigated through the kinetic resolution of racemic substrates. These studies are supported by kinetic analysis and quantitative rate accelerations that provide an improved understanding of host structure on a chemoselective and stereochemically complex reaction.

## Results and discussion

### Effects of host structure on product selectivity

Differences in selectivity were examined by varying the structures of both hosts and substrates. It has been established that host catalysts often exhibit strict substrate selectivity based on guest size.^34,35^ Following this precedent, the interaction of host and substrate size was tested by examining the effect of increasing host cavity volume on reaction stereoselectivity. Earlier reports have documented the difficulty of preparing pyrene-core host (±)-**3** in the absence of a strongly-bound template.^36^ However, treatment of reaction mixtures containing appropriate metal and ligand components with KOH and acetone allowed for the isolation of (±)-**3** and **4**, in analogy with the procedure reported for the preparation of solvent-occupied (±)-**1**.^37^ Previously, Δ_4_-**1** mediated enantioselectivities of up to 78% in the aza-Cope rearrangement of enammonium cations and 69% in intramolecular Prins reactions were observed, a result which attests to the potentially high degree of enantiodifferentiation between Δ_4_-**1** and catalytically relevant substrates.^31,38^ These examples of molecular recognition have been attributed to predominantly steric and π-interactions, as chiral induction is thought to proceed through contact of guest with naphthalene spacers. Given this precedent, the investigations reported herein were focused on a class of substrates that differ in their alkyl substituents at the β-position but are otherwise identical with regard to functional groups. This modification was aimed at avoiding the introduction of additional functional groups^39–41^ in the substrate that could dramatically alter the mechanism or stability of these compounds. Toward this end, terpene derivatives **5a–c** were separately treated with catalysts in either pure phosphate buffer solution or MeOD-*d*_4_/100 mM phosphate buffer cosolvent. After heating, the organic portions of the reaction mixtures were extracted and product distributions measured by ^1^H NMR integration. During these trials product ratios were found to be insensitive to moderate changes in cosolvent composition, temperature, time and pD.

Initially, differences in product selectivity resulting from the choice of host chelator were examined by comparing product mixtures following treatment of various substrates with (±)-**1** and **2**. The extent to which enantiopure **2** distinguishes between enantiomers (*S*)-**5a** and (*R*)-**5a** was tested. Note that this experiment is not possible with resolved (±)-**1** due to the presence of residual NMe_4_^+^, which inhibits catalysis.^31,42,43^ Product ratios varied between these treatments, with a higher *trans* selectivity observed between Δ_4_-**2** and (*R*)-**5a** (*trans*/*cis*: 69/31, entry 3a) than with Δ_4_-**2** and (*S*)-**5a** (*trans*/*cis*: 48/52, entry 2a). The averaged product *trans*/*cis* selectivity resulting from these treatments (58/42) is similar to that resulting from treatment of racemic **5a** with host (±)-**1** (65/35, entry 1a). In order to achieve similar levels of conversion under otherwise identical conditions, it was necessary for catalyst loadings of Δ_4_-**2** to vary by a factor of two between treatments of (*R*)-**5a** and (*S*)-**5a**, an observation which suggests a moderate degree of recognition between Δ_4_-**2** and enantiomers of **5a**. Likewise, *trans*/*cis* ratios were within error between treatments of (±)-**1** or Δ_4_-**2** with **5c**, although a difference in selectivity between (±)-**3** and Λ_4_-**4** (entries 4a and 5a) with substrate (±)-**5a** was observed for reasons that are unclear. Nonetheless, in the majority of trials completed, varying the host chelator did not affect product selectivities by more than a small margin.

**Scheme 1:**
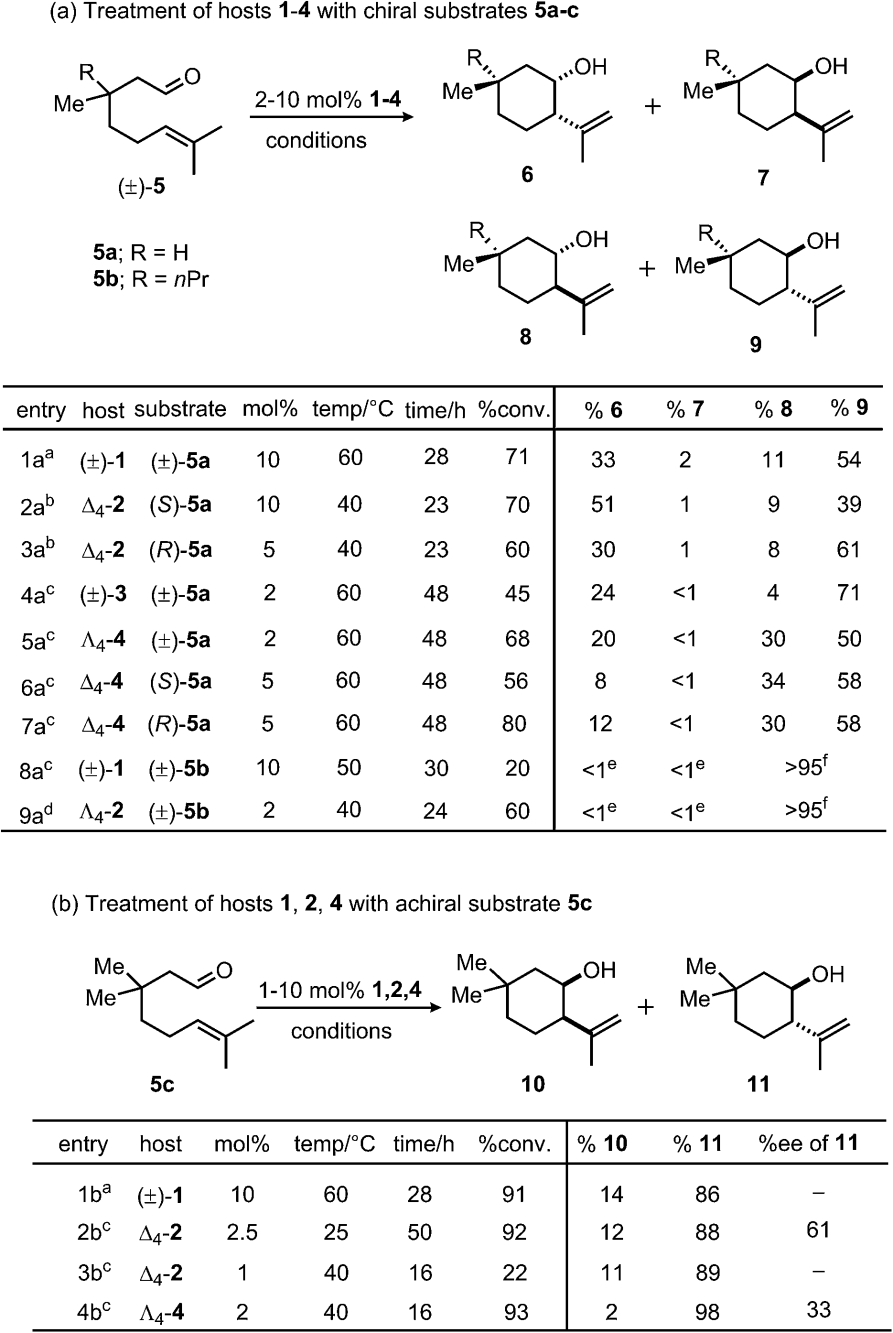
General conditions effective in the cyclizations of (a) chiral substrates **5a–c** and (b) achiral substrate **5c** with catalysts **1–4**. ^*a*^50 mM phosphate buffer, pH 7.50; ^*b*^100 mM phosphate buffer, pD 8.00; ^*c*^1 : 1 MeOD-*d*_4_/100 mM phosphate buffer, pD 8.00; ^*d*^1 : 1 MeOD-*d*_4_/100 mM phosphate buffer, pD 5.00; ^*e*^product not observed by ^1^H NMR spectroscopy or GC-MS. ^*f*^A single trans product was exclusively formed; the relative stereochemistry at the 1-position (–Me/–*n*Pr) could not be unambiguously determined. Selectivity measurements have an estimated error of ≤3%.

Next, product distributions from naphthalene-based catalysts were compared to those resulting from treatment with larger pyrene analogues (±)-**3** and **4**. In contrast to the selectivity observed from catalysis by Δ_4_-**2**, treatment of **5c** with pyrene-based Λ_4_-**4** resulted in the rapid formation of *trans* product with high selectivity (*trans*/*cis*: 98/2: entry 4b), demonstrating that increasing host cavity size through the use of a larger spacer can enhance stereoselectivity for *trans* products. In contrast, the high *trans* selectivities in entries 8a and 9a reflect the stereochemical preference of substrate **5b** due to a substantial 1,3-diaxial repulsion. When corrected for catalyst concentration, cyclization of **5c** by Λ_4_-**4** proceeds more efficiently than that of Δ_4_-**2** based on pseudo-first-order fits to the levels of conversion presented in Scheme 1 (*k*_rel_ ≈ 5; ΔΔ*G* ^‡^ = 1 kcal mol^–1^). This preference for *trans* products was also observed between treatment of (±)-**5a** with naphthalene host (±)-**1** and pyrene analogues (±)-**3** and Λ_4_-**4** (entries 1a, 4a and 5a). From these results, it is clear that exchanging a naphthalene for a pyrene spacer can affect a change in product diastereoselectivity that is consistent among different substrates (**5a** and **5c**), as well as different hosts ((±)-**3**, Λ_4_-**4**).

Collectively these observations suggest that the nature of chelator (CAM or TAM) used has little effect on the diastereoselectivity of this reaction. An exception to this trend results when the enantiopure hosts Δ_4_-**2** or Δ_4_-**4** interact in a diastereomeric fashion with substrate enantiomers (*i.e*. (*S*)- and (*R*)-**5a**). In contrast, the choice of spacer has a clear effect on product distributions, as is apparent from product selectivities resulting from treatment of **5a–c** with hosts (±)-**1** and **2** compared to (±)-**3** and **4**. Generally, it was observed that *trans* selectivity increases with cavity size, which may also accompany an improvement in catalytic efficiency. Consistent with these observations, gas-phase DFT calculations. Suggest that the barrier for the cyclization of **5a** is slightly lower for the transition state leading to the major *trans* product compared to that leading to the corresponding *cis* product.^40^ Based on these results, it is likely that the constrictive cavities of (±)-**1** and **2** may destabilize the transition state leading to *trans* products, an effect that also results in higher selectivity for *cis* products relative to analogous reactions in larger hosts (±)-**3** and **4**.

### Effect of host and guest size on enantioselective catalysis

The relationship between guest volume and catalyst enantioselectivity was investigated in the kinetic resolution of chiral starting material. In a catalytic kinetic resolution, the relative reaction rates of substrate enantiomers can be expressed as a selectivity factor (*s*; eqn (1)),^44^ which is determined by ΔΔ*G* ^‡^ between diastereomeric transition states. It was hypothesized that if the size of substrates was increased, an increase in *s* may be observed due to increased steric interactions between encapsulated substrate and the aromatic walls of Λ_4_-**2**, which are presumably the surfaces that induce enantioselectivity in these reactions.^31,38^

Toward this end, selectivity factors were measured for chiral starting materials **5a** and **5b** (1 : 1 MeOD-*d*_4_/100 mM phosphate buffer cosolvent, pD 5.00, 25 °C). While Λ_4_-**2** exhibited low chiral discrimination for **5a** (*s* = 1.8; ΔΔ*G* ^‡^ = 0.35 kcal mol^–1^), selectivity increased for larger substrate **5b** (*s* = 4.45; ΔΔ*G* ^‡^ = 0.88 kcal mol^–1^). Following this observation, product ee's resulting from the cyclization of **5c** with hosts Δ_4_-**2** and Λ_4_-**4** were compared. In the latter case, an analogous trend was observed; product enantioselectivity was greater when a smaller cavity was used (entry 2b, 61% ee; ΔΔ*G* ^‡^ = 1.14 kcal mol^–1^; Scheme 1)^31^ relative to a larger one (entry 4b, –33% ee; ΔΔ*G* ^‡^ = 0.56 kcal mol^–1^; Scheme 1). In further support of this notion, a smaller degree of recognition (indicated by small differences in product selectivity and conversion) was observed between enantiomers of **5a** and host Δ_4_-**4** (entries 6a and 7a) compared to analogous trials with smaller host Δ_4_-**2** (entries 2a and 3a). While these observations are consistent with the notion that the magnitude of host-mediated enantioinduction increases with guest size (or decreases with host cavity size), it should be noted an analogous trend was not observed between two previously reported achiral Prins substrates.^31^
1
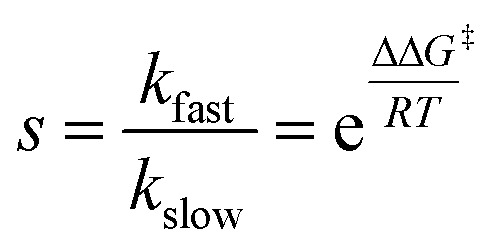



### Mechanistic considerations

**Scheme 2:**
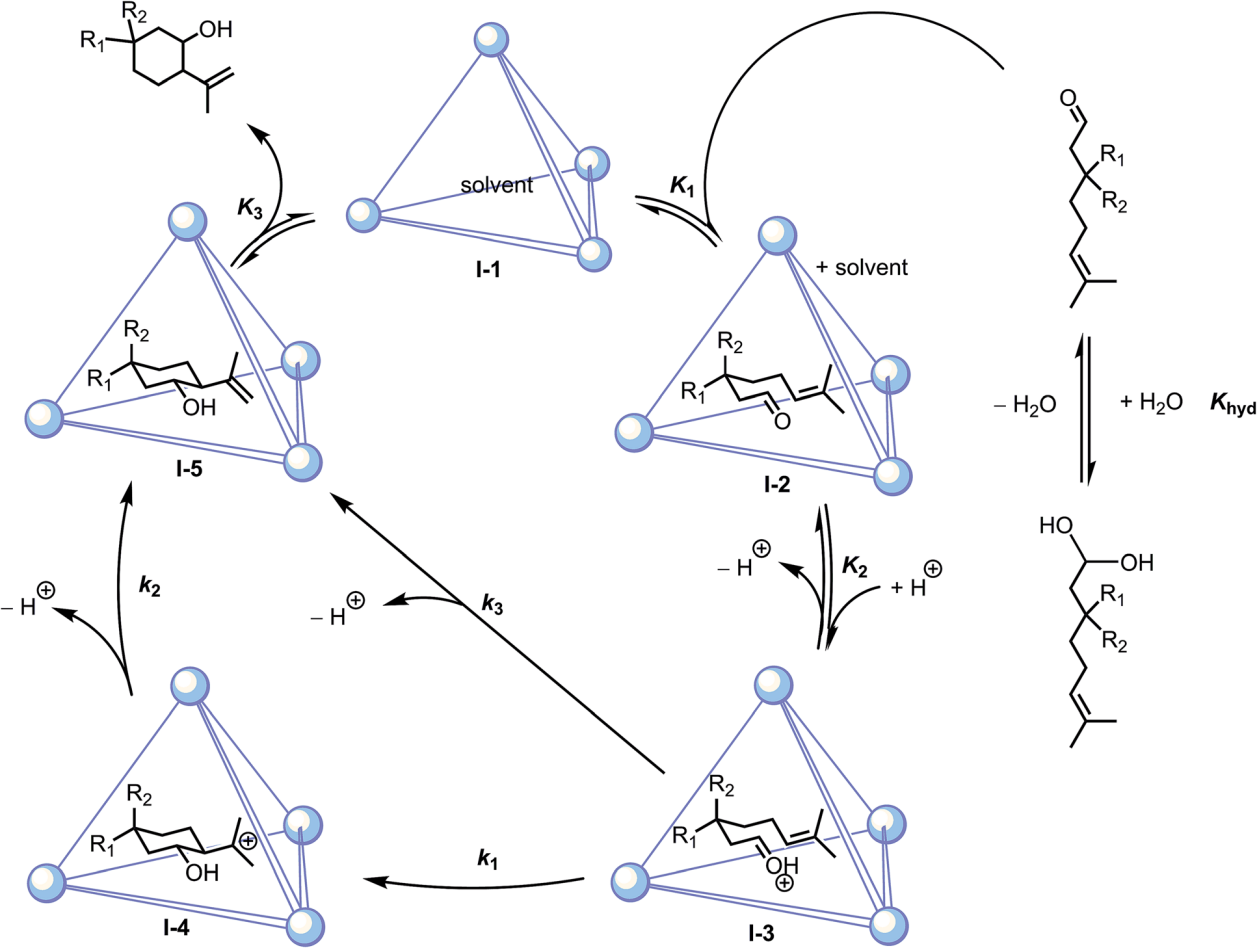
Proposed mechanisms for host-catalyzed Prins cyclizations, where stepwise (*k*_1_, *k*_2_) or concerted (*k*_3_) pathways are plausible.

In order to determine the role of catalyst, substrate and bulk solution acidity on reaction rate, the order in (±)-**1**, **5c** and D^+^ were determined using initial rate measurements (^1^H NMR spectroscopy). Because this reaction proceeds initially by a reversible encapsulation pre-equilibrium, saturation of catalyst by substrate is possible in principle. In practice, however, saturation by **5c** was not observed due to the low affinity of this substrate for (±)-**1** or **2** and the limited solubility of substrate in MeOD-*d*_4_/phosphate buffer cosolvent. Consequently, the rate of reaction was measured to be first-order in (±)-**1** as well as substrate **5c**. In contrast, a 0.4(1)-order dependence was measured between *k*_obs_ and D^+^ over the pD range 6.9–8.0. Taken together, these experiments demonstrate that the (±)-**1**-catalyzed cyclization of **5c** obeys the empirical rate law: rate = *k*_obs_[substrate][host][D^+^]^0.4(1)^, which at constant pD reduces to rate = *k*_obs_[substrate][host]. These measurements and subsequent observations described below are consistent with the mechanisms proposed in Scheme 2.

## Investigation of the catalytic steps

### Aldehyde-hydrate (*K*_hyd_) and encapsulation (*K*_1_) pre-equilibria

Under aqueous conditions, aldehyde-containing substrates underwent reversible hydration, a process which was observed by ^1^H NMR spectroscopy. Evidence for the assignment of the hydrate was obtained by varying the proportion of MeOD-*d*_4_ to phosphate buffer cosolvent. While the hydrate C–*H* resonance was absent in pure MeOD-*d*_4_, the ratio of hydrate to aldehyde integrals increased with increasing proportion of aqueous phosphate buffer. During these experiments, the sum of aldehyde to hydrate resonances remained constant and was equal to the sum of corresponding alkene C–*H* resonances. Extraction of this mixture into CDCl_3_ and subsequent analysis by ^1^H NMR spectroscopy resulted in the quantitative recovery of aldehyde, confirming that hydration is reversible. The magnitude of the aldehyde-hydrate ratio also varied considerably between substrates; the ratio of aldehyde to hydrate was lower for less hydrophobic substrates in aqueous solution.^45^ Following these qualitative experiments, it was next investigated whether substrate-dependent *K*_hyd_ pre-equilibria could affect guest binding and catalysis.

To determine the effect of encapsulation on aldehyde-hydrate equilibria, homogenous solutions of (±)-**5a** and **5c** were treated with host (±)-**1**. ^1^H NMR analysis revealed significant broadening of aldehyde C–*H* resonances, indicating guest exchange.^33,46,47^ In contrast, hydrate resonances underwent no such broadening, indicating a negligible degree of encapsulation between hydrates of (±)-**5a**, **5c** and (±)-**1**. This result is attributable to the higher solvation of hydrate compared to aldehyde in the aqueous cosolvent employed. Increasing the ratio of (±)-**1** to **5c** resulted in an increase in the integrals of the encapsulated aldehyde resonances, accompanied by a decrease in the integral of the corresponding unencapsulated aldehyde resonance and R(OH)_2_C–*H* hydrate resonance. These results confirm that encapsulation perturbs the aldehyde-hydrate equilibrium by selective encapsulation of aldehyde over hydrate.

It was hypothesized that the encapsulation of substrate aldehyde is driven by the hydrophobic effect, a process that has been shown to be controlled entropically by the release of encapsulated solvent.^48,49^ In order to examine this effect, the influence of organic cosolvent on catalysis was investigated. Previously, the use of organic cosolvents had been observed to inhibit the (±)-**1**-catalyzed hydrolysis of orthoformates, an effect which results from the lower affinity of host and guest in nonaqueous solvents due to attenuation of the hydrophobic effect.^46,50,51^ In contrast to purely aqueous conditions where the appearance of broad upfield resonances confirms a comparably high degree of guest association, guest binding is attenuated in a 1 : 1 (v/v) aqueous phosphate buffer/MeOD-*d*_4_ cosolvent. Under homogenous conditions, encapsulated aldehyde, unencapsulated aldehyde and total host concentrations were measured against an internal standard, from which dissociation constants were calculated (see *K*_M_ values, Table 1).

**Table 1:** Kinetic parameters for host-catalyzed Prins reactions^*a*^

Entry	Substrate	Catalyst	*K*_M_ (mM)	*k*_cat_ (s^–1^)	*k*_cat_/*K*_M_ (M^–1^ s^–1^)	(*k*_cat_/*K*_M_)/*k*_uncat_ (M^–1^)	*k*_cat_/*k*_uncat_
1	**5c**	(±)-**1**	5.4 × 10^2^	8.9 × 10^–4^	1.6 × 10^–3^	2.9 × 10^4^	1.6 × 10^4^
2	**5c**	Λ_4_-**2**	5.8 × 10^2^	5.4 × 10^–3^	9.3 × 10^–3^	1.6 × 10^5^	9.5 × 10^4^
3	(±)-**5a**	(±)-**1**	2.0 × 10^2^	5.5 × 10^–4^	2.7 × 10^–3^	2.5 × 10^5^	5.0 × 10^4^
4	(*S*)-**5a**	Δ_4_-**2**	3.3 × 10^2^	1.0 × 10^–3^	3.0 × 10^–3^	2.8 × 10^5^	9.1 × 10^4^
5	(*R*)-**5a**	Δ_4_-**2**	1.8 × 10^2^	2.1 × 10^–3^	1.2 × 10^–2^	1.1 × 10^6^	1.9 × 10^5^

^*a*^ *k*_uncat_ for **5c**: 5.7(6) × 10^–8^ s^–1^; (*S*)-**5a**: 1.1(1) × 10^–8^ s^–1^; *K*_M_ measurements have an estimated error of 10%; conditions for all runs: 1 : 1 MeOD-*d*_4_/100 mM phosphate buffer, pD 8.00; 25 °C.

In spite of the low magnitudes of association between host and substrate while using this cosolvent, catalysis in a 1 : 1 MeOD-*d*_4_/phosphate buffer proceeded with an efficiency similar to that observed under pure aqueous buffer conditions. Catalysis did not proceed in pure methanol under otherwise identical conditions. The maintenance of catalytic efficiency in this cosolvent can be attributed in part to the higher concentrations of soluble guest in homogenous solutions, which, to some extent, offsets the lower degree of association observed. These experiments collectively suggest that host-catalyzed Prins cyclizations proceed initially through the displacement of solvent from the host cavity by substrate, an event that is driven by the hydrophobic effect.

### Protonation of aldehyde oxygen (*K*_2_), nucleophilic capture by alkene (*k*_1_) and proton elimination (*k*_2_); concerted pathway (*k*_3_)

After encapsulation, we propose that the host activates the substrate by stabilization of its conjugate acid, driving protonation of the carbonyl oxygen, followed by intramolecular nucleophilic attack by the pendant alkene. In principle, protonation of the carbonyl could occur prior to encapsulation. However, the latter scenario seems unlikely based on prior studies where the catalytic resting states for (±)-**1**-mediated orthoformate hydrolysis and Nazarov cyclizations were identified as the neutral guest species, whose ether- and alcohol-based oxygen atoms have a basicity similar to those of carbonyl oxygen functionalities.^33,52^ To examine equilibrium *K*_2_, the rate of cyclization of **5c** by (±)-**1** was measured to be slightly nonlinear between pD 6.9 and 8.0. Over this range, the dependence of *k*_obs_ on bulk solution was approximately 0.4(1)-order. This result bears analogy to the 0.5(1)-order relationship between *k*_obs_ and pD previously measured in the **1**-catalyzed Nazarov cyclization.^52^ Because the aldehyde-hydrate equilibrium was perturbed slightly toward aldehyde at lower pD in the Prins reactions, the less than first-order dependence could not be due to a bulk solution effect on this pre-equilibrium.^53–55^ These observations suggest that host-catalyzed Prins reactions are only indirectly promoted by increasing acidity of the bulk solution, a result that is inconsistent with a mechanism that proceeds exclusively through specific acid catalysis by D_3_O^+^. General acid catalysis could be operative, wherein the changes in *k*_obs_ with D^+^ may correlate with the p*K*_a_ of a general acid involved in catalysis. In principle, a gallium-bound catecholamide functionality could act as a general acid catalyst in this regard.^56,57^ These measurements suggest that host-catalyzed Prins reactions are promoted by bulk solution acidity, albeit in a complex manner.

Previous investigations have demonstrated that (±)-**1** can enforce a chair conformation of acyclic guests.^51,58,59^ Based on this precedent, it is likely that cyclization is accelerated by steric constraints afforded through encapsulation. Following protonation of the encapsulated substrate, cyclization could conceivably proceed through either a step-wise (*k*_1_, *k*_2_) or concerted (*k*_3_; *cf.* Scheme 2) pathway. These mechanisms could, in principle, be distinguished by the direct observation of carbocation **I-4** (Scheme 2). However, under the catalytic conditions employed, guest binding is too weak to permit the definitive characterization of this possible species. To address whether a stepwise or concerted pathway was likely operative in host-catalyzed cyclizations, prior mechanistic investigations of **5a** cyclizations were consulted. Under anhydrous Lewis acidic conditions, the cyclization of **5a** is thought to proceed through a concerted mechanism and results in *trans* or *cis* products, depending on the nature of the catalyst.^59–61^ In contrast, Brønsted acid-catalyzed cyclizations proceeding under either aqueous or anhydrous conditions have been shown to afford predominantly *cis* products resulting from nucleophilic capture of carbocation **I-4**.^40,62–65^ It has been suggested that the *cis* selectivity in the latter cases results from ion pairing with carbocation **I-4**, which would stabilize this intermediate, leading to products of nucleophilic capture by water or anion.^64^ In support of this notion, a Lewis acid catalyst reported by Kočovský *et al.* afforded ene products under anhydrous conditions, but diols were observed when trace amounts of water were present in the reaction mixture.^66^ DFT calculations have also suggested that the preference for *trans* over *cis* ene products for **5a** cyclization decreases when moving from concerted to stepwise mechanisms.^40^ These studies suggested to us that the presence of water or an appropriate anion could, by stabilizing a carbocation intermediate, influence not only product chemoselectivity, but diastereoselectivity as well.

Based on these reports, it was unclear whether the exclusion of water during host-catalyzed Prins cyclizations of **5a** could explain the observed *trans* product diastereoselectivity. This stereoselectivity is unlikely to have resulted from constrained steric interactions, as increased steric confinement has been shown to accompany increased *cis* product selectivity (see Scheme 1). To probe whether a low concentration of water in the host cavity could account for the observed *trans* diastereoselectivity, **5a** was treated with various MeOH/100 mM aqueous phosphate buffer (pH 3.20) cosolvents in the absence of host, where the volumetric ratio of MeOH to buffer varied between 0 and 1.5. After heating, products were extracted and analyzed by ^1^H NMR spectroscopy. While the proportions of alkene products formed from these treatments were minor (10–20% product selectivity), the *trans* selectivity of these products increased monotonically with an increasing proportion of MeOH (at 0% MeOH, *trans*/*cis* = 0.84; at 60% MeOH, *trans*/*cis* = 1.56). We speculate that this correlation between an increasing ratio of MeOH and *trans* product selectivity results from the destabilization of a stepwise cyclization mechanism in the presence of a lower dielectric bulk cosolvent. In principle, this change in mechanism could be accomplished through lowering the effective concentration of water in bulk solution, the absence of which would conceivably destabilize the stepwise transition state leading to *cis* products. Based on these experiments, it is possible that host-catalyzed cyclizations may be more concerted in character than stepwise processes occurring under conventional Brønsted acid catalysis. Furthermore, simply the exclusion of water from the host cavity during catalysis could account for the observed *trans* product selectivity.

### Product displacement and turnover (*K*_3_)

In order to test whether cyclization was reversible under the reaction conditions employed, a mixture of alkene products (±)-**6**–**9a** was treated with an aqueous solution of (±)-**1** (7 mM, 8 mol%) and subjected to heating. After extraction, no starting material (±)-**5a** or changes in product distribution were observed, suggesting that catalysis is irreversible under catalytically relevant conditions.

Although product inhibition is a common challenge in cavity-mediated catalysis,^67–73^ no deviation from first-order kinetics was seen through 90% conversion of **5c** with 4 mol% Λ_4_-**2**, an observation which demonstrates that inhibition is largely negligible and implies that *K*_1_ > *K*_–3_. It was hypothesized that the absence of product inhibition results from the high solvation of product alcohol by the bulk solution. In this instance, the higher degree of product solvation compared to that of the starting material could provide a driving force for turnover. Consistent with this notion, the water solubility of **5a** (0.9 mM) is lower than that of (–)-menthol (4.0 mM) by a factor of 4.4.^74^ In order to test the effect of the alcohol functionality on encapsulation and catalysis, a stock solution of (±)-**1** was partitioned to two identical reaction flasks which were treated with an equimolar stock solution of either (–)-menthol or (–)-menthyl chloride in an aqueous methanolic cosolvent. ^1^H NMR analysis revealed that, although encapsulation was evident for (–)-menthyl chloride, no encapsulation was observed for (–)-menthol. This observation attests to the importance of alcohol hydrogen bonding in guest solvation and consequently, host–substrate affinity. These mixtures were treated with equal concentrations of (±)-**5a** and heated, after which the organic portions were extracted and first-order rate constants determined based on the level of conversion the starting material had undergone. From these measurements, a small but measurable degree of catalytic inhibition was observed for the (–)-menthyl chloride case relative to the condition with (–)-menthol (*k*_rel_ = 0.7(1)). Consequently, it is conceivable that the formation of alcohol-containing products from aldehyde-containing starting materials drives turnover through a preferential hydrogen bonding interaction between product and aqueous solvent, a trait that correlates with the greater solubility of alcohol-containing products compared to aldehyde-containing starting materials. Combined with the high thermal persistence of **2**, this catalytic property allows for high turnover numbers to be achieved. Under dilute conditions (0.049 mM, 0.045 mol% Λ_4_-**2**), catalysis proceeded with up to 840 turnovers over two weeks, which is among the highest reported for intramolecular Prins or carbonyl-ene cyclizations.^60,75–79^

### Michaelis–Menten analysis, rate accelerations

The observation that host–substrate complexes undergo fast chemical exchange,^80^ accompanied by a relatively slow rate of catalysis, implies a mechanism involving a fast pre-equilibrium including encapsulation followed by a rate-limiting process that was irreversible under the reaction conditions used (eqn (2)). Based on these characteristics, guest-binding and subsequent catalytic steps were deconvoluted with Michaelis–Menten analysis. As mentioned previously, the cyclization of **5c** was measured to be first-order in substrate and (±)-**1**, both of which are consistent with the rate law given below (eqn (3)).^81,82^
2
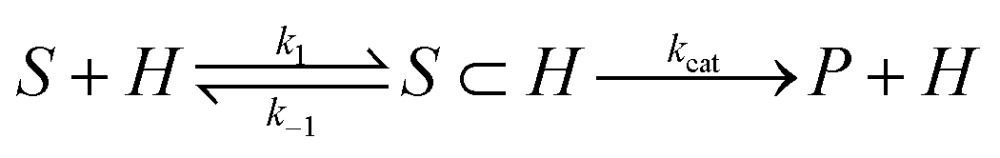

3
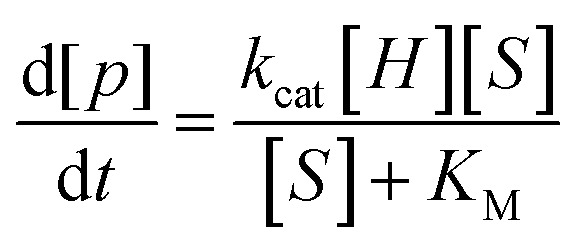

4
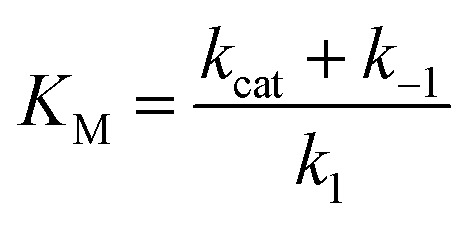



Because guest exchange is fast with respect to cyclization, experimentally determined *K*_d_ = *K*_M_. Uncatalyzed cyclizations proceeded slowly; less than fifteen percent of starting materials (*S*)-**5a** and **5c** were observed to cyclize over the course of four weeks. Nonetheless, these low levels of conversion were sufficient to quantify the uncatalyzed rates of reaction in bulk solution. The experimental *k*_uncat_ for **5a** is roughly an order of magnitude faster than the calculated gas-phase value, a difference which can be accounted for by the stabilizing role of water on the calculated transition state.^83^ Notably, the uncatalyzed cyclization of **5c** proceeds approximately five times faster than that of (*S*)-**5a**. The magnitude of this difference in rate is small compared to other examples of the *gem*-disubstituent effect.^84^ In contrast, catalysis by **1–4** proceeds relatively quickly, with half-lives on the order of hours to a day, depending on the experimental conditions. Specificity factors (*k*_cat_/*K*_M_) were largest for (*R*)-**5a** and **5c** with host **2**. These properties are a reflection of (a) the generally higher rate of catalysis with **2** compared to (±)-**1**, (b) the slightly more hydrophobic nature of **5c** compared to **5a**, which favors encapsulation both through the hydrophobic effect and aldehyde-hydrate equilibria, and (c) the complementarity of the (*R*)-**5a**/Δ_4_-**2** diastereomeric pairing in catalysis. Catalytic proficiencies ((*k*_cat_/*K*_M_)/*k*_uncat_), as measures of transition state stabilization afforded through encapsulation relative to the uncatalyzed reactions, were consistently higher for less hydrophobic substrates (*R*)-, (*S*)- and (±)-**5a**. This trend is a reflection of the tendency for host to drive substrate cyclization through the selective encapsulation of aldehyde over hydrate. In the case of (*S*)-**5a**, *k*_uncat_ is low in part due to the relatively hydrate-favored aldehyde-hydrate equilibrium. The tendency for hosts to compensate for the *gem*-disubstituent effect also likely contributes to this trend.^24^ Catalytic rates (*k*_cat_) for all substrates were consistently higher in **2** than (±)-**1** by less than an order of magnitude. On the whole, rate accelerations ranged between 10^4^ and 10^5^ (5.7–7.2 kcal mol^–1^), the latter of which are among the largest observed with a synthetic supramolecular cavity.^69,85–90^

## Conclusion

While variation in the host chelator was generally observed to produce no significant changes in product selectivity, catalysis in TAM-based **2** proceeded with consistently higher efficiency than CAM-based (±)-**1**. In contrast, variation in host spacer (Nap or Pyr) resulted in changes in efficiency and product selectivity. Up to 840 turnovers were observed, which numbers among the highest known for intramolecular Prins cyclizations. Rate accelerations for the catalyzed reactions are on the order of 10^4^–10^5^ relative to uncatalyzed treatments, which are likewise among the highest reported in the field of host–guest catalysis. The trends reported herein enable a better understanding of enzyme-mimic microenvironment in the context of chemo-, diastereo- or enantioselective catalysis. In a broader sense, this work aims to build a fundamental understanding of biological catalysts using simple synthetic models.

## Experimental procedures

Unless otherwise noted, reactions and manipulations were performed using standard Schlenk techniques or in an oxygen-free wet box under nitrogen atmosphere. All solvents were degassed under nitrogen for 20 min before use. Glassware was dried in an oven at 150 °C overnight or by flame before use. Column chromatography was carried out on a Biotage SP1 MPLC instrument with prepacked silica gel columns. NMR spectra were obtained on a Bruker AV 400 (400 MHz), AV 500 (500 MHz) or AV 600 (600 MHz) spectrometer. Chemical shifts are reported as *δ* in parts per million (ppm) relative to residual protiated solvent resonances. NMR data are reported according to the format s = singlet, d = doublet, t = triplet, m = multiplet, b = broad; integration; coupling constant. Mass spectral data were obtained at the QB3 Mass Spectrometry Facility operated by the College of Chemistry, University of California, Berkeley. Electrospray ionization (ESI) mass spectra were recorded on a Finnigan LTQ FT mass spectrometer. Chiral GC analyses were conducted using a HP 6850 series GC system fitted with a chiral column, BetaDex 120 Fused Silica Capillary Column (30 m × 0.25 mm × 0.25 um film thickness). Unless otherwise noted, chemicals were obtained from commercial suppliers and used without further purification. Preparations of **1**, **2** and **5c** have been described.^24,31^ Compound **3** was prepared by the route previously reported^36^ and precipitated with acetone. Unless otherwise noted, reported pD values are uncorrected for the glassy electrode artifact (*i.e.* pD_corr_ = pD_read_ + 0.40).^91^

### General preparation of (**6–11**) used for selectivity determination and ee determination of starting material and product

This procedure is adapted from earlier reports.^24,31^ Aldehyde starting material (45 μmol), **1**, **2**, **3** or **4** (4.2 μmol), 250 μL MeOD-*d*_4_ and 250 μL phosphate buffer (for example, 100 mM K_2_DPO_4_, pD 8.00) were added to a standard NMR tube. This slightly heterogeneous mixture was heated in an oil bath for a period of time as indicated, after which the organic components were extracted (3 × 300 μL CDCl_3_) and passed through a pipet containing a thin filter of glass fiber. Selectivity and conversion were determined by ^1^H NMR integration. *Cis* and *trans* product isomers were differentiated by characteristic alcohol C–*H* coupling^24^ and accompanying alkene C–H resonances (*ca.* 4.9 ppm). GC-MS analysis of these samples confirmed the presence of only aldehyde starting material and alkene products. Products and starting material were isolated by silica gel chromatography (10% EtOAc in hexanes) prior to ee determination by chiral-GC and characterization. Selectivity factors were determined by direct rate measurement ((*R*)- and (*S*)-**5a**) or based on conversion and ee (**5b**), as described in ref. 44. Turnover was assessed following treatment of Λ_4_-**2** (7.0 mg, 0.0015 mmol) with **5c** (540 mg, 3.2 mmol) in 1 : 1 MeOH/100 mM phosphate buffer, pH 5.40 (30 mL, 13 days 2 h, 50 °C) and this heterogeneous mixture stirred vigorously, after which organic portions were extracted (3 × 30 mL DCM), dried over MgSO_4_, solvent removed *in vacuo* and the combined yield of **10** and **11** assessed using an internal standard of mesitylene in CDCl_3_ (38% yield, 1.2 mmol). Due to the elevated temperature and long reaction time employed, a modest amount of background reactivity was evident in the production of *p*-menthane-3,8-diols (∼10% yield).^24^ Treating a higher concentration of Λ_4_-**2** (0.30 mM) with 530 equivalents of **5c** afforded 455 turnovers after 3 d and only trace (<1% yield) *p*-menthane-3,8-diols. Characterization of *trans*-5-methyl-2-(prop-1-en-2-yl)-5-propylcyclohexan-1-ol (**8**/**9b**): ^1^H NMR (600 MHz, CDCl_3_): *δ* (ppm) 4.91 (s, 1H), 4.85 (s, 1H), 3.64 (dt, 1H), 1.90–1.76 (m, 2H), 1.75 (s, 3H), 1.35–1.45 (m, 2H), 1.23–1.35 (m, 6H), 1.09 (t, 2H), 0.90 (s, 3H), 0.85 (t, 3H); ^13^C NMR (151 MHz, CDCl_3_): *δ* (ppm) 146.96, 112.63, 67.76, 54.88, 48.93, 45.08, 36.92, 35.45, 25.58, 21.91, 19.07, 16.34, 14.49; HRMS (FTMS ESI) calculated for C_13_H_24_O: 196.1827; found: 196.1824.

### General procedure for rate measurements

In a typical experiment, a homogenous solution of aldehyde starting material (20 μmol), host **1** or **2** (2 μmol), 250 μL MeOD-*d*_4_ and 250 μL 100 mM phosphate buffer (from K_3_PO_4_/HCl; pD 5.00 or 8.00) was prepared and added to a standard NMR tube. The tube was then inserted in a preheated NMR probe (25.0(1) °C) within three minutes of its preparation and the reaction followed with single scan ^1^H experiments. In the case of more slowly reacting substrate/host pairs, reactions were monitored every 1–2 h over the course of 8–10 h, from which initial rates (*k*_obs_/mM s^–1^) were obtained. From these observed rates, *k*_cat_ values were calculated using initial substrate and host concentrations during catalysis and *K*_d_, which was substituted for *K*_M_ in eqn (2) in accordance with established procedure.^46,47^ Due to the low affinity of host and guest, *K*_d_ values were obtained separately using higher concentrations of host (*ca.* 15 mM). Based on the observation that only aldehyde species (not hydrate) were encapsulated, the effective aldehyde concentration at the beginning of the run was treated as [*S*] in eqn (3). Quantitative mass balances of product and starting material were observed during kinetic trials. Background rates (*k*_uncat_/s^–1^) of cyclization were obtained by following an analogous procedure where solutions were monitored every 1–2 days over the course of a month. During initial trials it was observed that many internal standards had an inhibitory effect on catalysis, which presumably results from (a) internal or external association of the standard to the host and (b) the low affinity of the host for substrate in the cosolvent used. Consequently, rates of product formation were referenced against the residual MeOH solvent resonance, whose concentration was confirmed at the end of the kinetic run by the addition of an internal standard of 3-(trimethylsilyl)-1-propanesulfonic acid, sodium salt. Rates were reproducible within 10% among identically prepared solutions.

### Preparation of **13**

The previously-reported carboxylic acid **12** (ref. 31) (100 mg, 0.32 mmol) was dissolved in DMF (4 mL), and DCC (80 mg, 0.39 mmol) and HOBt (52 mg, 0.39 mmol) were added to the solution. The solution was stirred at ambient temperature for 30 minutes, and 1,6-diammoniumpyrene hydrogen sulfate (123 mg, 0.290 mmol) was added in one portion, followed by triethylamine (180 μL). The dark brown solution was stirred at ambient temperature for 16 h. A white solid was filtered off, and to the resulting solution was added H_2_O (20 mL), forming a yellow precipitate. The suspension was filtered and washed with H_2_O (3 × 2 mL). The precipitate was extracted with methylene chloride (25 mL) and the solvent evaporated to afford a yellow powder (**13**) that was used without further purification in the next step.

### Preparation of **15**

To a solution of carboxylic acid **12** (148 mg, 0.48 mmol) in methylene chloride (7 mL) at 0 °C was added thionyl chloride (0.4 mL). The reaction mixture was stirred at 0 °C for 2 h. After that time, volatile materials were removed *in vacuo* to afford a tan oil. Methylene chloride (2 × 2 mL) was added and evaporated to afford the acid chloride **14** as a colorless powder, which was used without further purification. **14** was added to a solution of **13** and triethylamine (0.180 mL, 1.28 mmol) in CH_2_Cl_2_ (7 mL) at ambient temperature, and the resulting yellow solution was stirred for 40 h at ambient temperature. This yellow solution was diluted with CH_2_Cl_2_ (50 mL) and washed with aqueous HCl (1 N, 2 × 20 mL), aqueous NaOH (1 N, 2 × 20 mL), and brine (1 × 20 mL), and dried over Na_2_SO_4_. Solvent was removed and the resulting yellow powder reprecipitated from CH_2_Cl_2_/hexanes to afford **15** (130 mg, 51%) as a yellow powder. ^1^H NMR (600 MHz, CDCl_3_) *δ* 10.71 (s, 2H), 8.91 (d, *J* = 8.4 Hz, 2H), 8.19 (d, *J* = 8.3 Hz, 2H), 8.15 (d, *J* = 8.3 Hz, 2H), 8.09 (q, *J* = 9.2 Hz, 4H), 8.02 (d, *J* = 8.4 Hz, 2H), 7.86 (d, *J* = 9.4 Hz, 2H), 4.25 (s, 6H), 4.17 (dq, *J* = 9.5, 6.8 Hz, 2H), 4.12 (s, 3H), 1.23 (d, *J* = 6.7 Hz, 6H), 1.04 (s, 18H). ^13^C NMR (151 MHz, CDCl_3_) *δ* 163.44, 162.66, 151.65, 151.48, 131.69, 131.42, 129.73, 128.39, 128.28, 127.12, 127.09, 125.58, 125.54, 122.61, 121.35, 118.79, 62.52, 62.12, 53.55, 34.45, 26.53, 16.28. HRMS (FTMS ESI) calculated for [C_48_H_54_N_4_O_8_]: 814.3942, found 837.3818.

### Preparation of **16**

To a suspension of **15** (82 mg, 0.10 mmol) in methylene chloride (4 mL) was added BBr_3_ (0.076 mL, 0.80 mmol). The yellow suspension instantly turned orange and was stirred at ambient temperature for 16 h. The suspension was then poured over ice and warmed to ambient temperature. The suspension was filtered to give a yellow solid that was suspended in water (10 mL). The yellow suspension was heated at reflux for 16 h and then cooled to ambient temperature. The mixture was filtered to afford **16** (55 mg, 72%) as a fine yellow powder. ^1^H NMR (500 MHz, DMSO-*d*_6_) *δ* 12.87 (s, 2H), 12.15 (s, 2H), 11.30 (s, 2H), 8.51 (d, *J* = 9.0 Hz, 2H), 8.46 (d, *J* = 8.4 Hz, 2H), 8.40 (d, *J* = 8.2 Hz, 2H), 8.28 (q, *J* = 9.3 Hz, 4H), 7.72 (d, *J* = 8.6 Hz, 2H), 7.65 (d, *J* = 9.4 Hz, 2H), 4.11–4.03 (m, 2H), 1.16 (d, *J* = 6.9 Hz, 6H), 0.95 (s, 18H). ^13^C NMR (126 MHz, DMSO-*d*_6_, adduct with DCU) *δ* 167.76, 167.03, 149.77, 148.63, 131.19, 128.71, 127.70, 125.29, 124.67, 124.44, 124.10, 121.42, 119.28, 118.19, 117.16, 52.52, 47.52, 34.67, 34.66, 33.34, 26.33, 26.29, 26.02, 25.32, 24.47, 15.34, 15.34. HRMS (FTMS ESI) calculated for [C_44_H_46_N_4_O_8_ – H]^–^: 757.3243, found 757.3239.

### Preparation of **4**

**Scheme 3:**
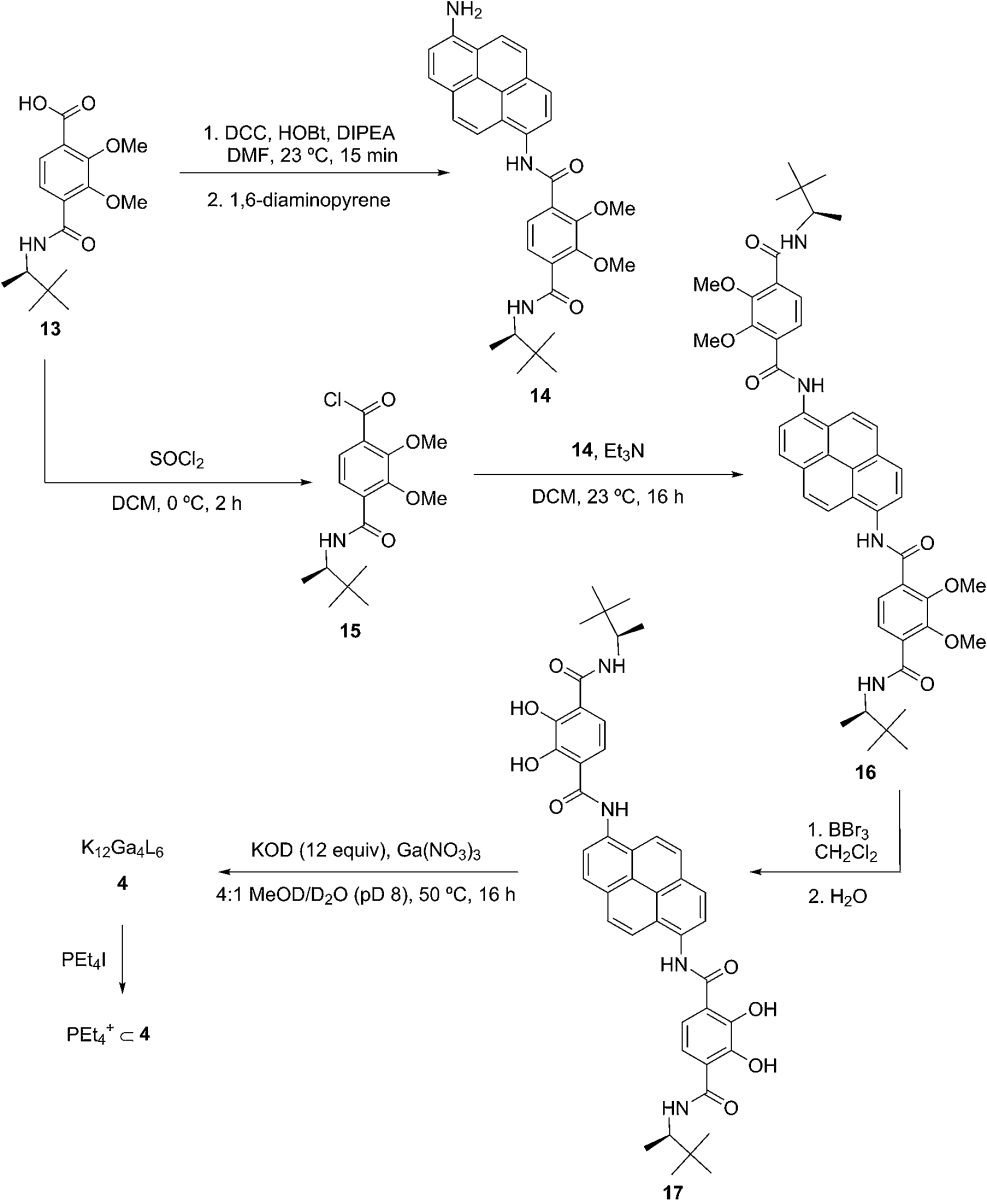
Preparation of host **4**. For simplicity, only one ligand enantiomer is shown.

In a glove box with a nitrogen atmosphere, KOD (3.52 mg, 0.064 mmol) was added to a suspension of **16** (24 mg, 0.032 mmol) in MeOD (0.64 mL), and the reaction mixture was stirred until the suspension became a homogeneous yellow solution. To this solution was added a 100 mM phosphate buffered solution of D_2_O at pD = 8.0 (0.16 mL) and Ga(NO_3_)_3_ (5.44 mg, 0.021 mmol). The reaction mixture was heated at 55 °C for 14 h and subsequently cooled to ambient temperature and filtered. Solvent was removed to form a yellow solid, which was recrystallized from MeOH/Et_2_O to afford **4** as a yellow solid (21 mg, 75%). ^1^H NMR (500 MHz, MeOD) *δ* 14.00 (br, N*H*), 8.90 (br, 2H), 8.28 (br, 2H), 7.56 (br, 4H), 7.17 (br, 2H), 6.98 (br, 2H), 4.52 (br, 12H), 1.06 (d, *J* = 6.4 Hz, 36H) 0.60 (s, 108H). Upon addition of PEt_4_I (5.5 mg, 0.02 mmol) to **4** in MeOD, encapsulation was observed within 15 minutes to afford PEt_4_^+^ ⊂ **4** as one species. ^1^H NMR (600 MHz, Methanol-*d*_4_) *δ* 14.30 (s, 7H), 11.59 (s, 12H), 9.09 (s, 12H), 8.49 (s, 12H), 7.80 (s, 12H), 7.65 (s, 12H), 7.26–7.20 (m, 12H), 7.09 (s, 12H), 4.60 (s, 12H), 1.14 (s, 81H), 0.68 (s, 115H), –3.10 to –3.25 (m, 20H). TOF ES MS: found [PEt_4_^+^ ⊂ H_6_M_4_L_6_]^5–^, [PEt_4_^+^ ⊂ PEt_4_H_6_M_4_L_6_]^4–^ (Scheme 3).

### Preparation of **5b**

Propylmagnesium chloride (9.80 mL, 9.80 mmol, and 1.0 M solution in ether) was added drop wise to a solution of CuI (1876 mg, 9.80 mmol) in dry ether at –10 °C. The reaction mixture was cooled to –78 °C and (*E*/*Z*)-3,7-dimethyl-oct-2-enal (1500 mg, 9.80 mmol) was added drop-wise at this temperature. The resulting mixture was stirred for 1 h and slowly brought to room temperature. The mixture was washed with saturated NH_4_Cl aqueous solution followed by brine solution and was extracted with ether. The combined organic layer was concentrated *in vacuo*. Flash chromatography on silica gel (10% ethyl acetate in hexanes eluant) afforded 130 mg (0.66 mmol, 7% yield) of the purified product 3-propyl-3,7-dimethyl-octanal **5b** as a yellow oil. ^1^H NMR (600 MHz, CDCl_3_): *δ* (ppm) 9.80 (t, 1H), 5.05 (t, 1H), 2.21 (d, 2H), 1.89 (q, 2H), 1.69 (s, 3H), 1.55 (s, 3H), 1.2–1.4 (m, 6H), 0.98 (s, 3H), 0.78 (t, 3H); ^13^C NMR (151 MHz, CDCl_3_): *δ* (ppm) 203.68, 131.45, 124.27, 52.79, 42.42, 40.02, 36.25, 25.62, 25.23, 22.24, 17.50, 16.75, 14.73; HRMS (FTMS ESI) calculated for C_13_H_24_O: 196.1827; found: 196.1830. Using two equivalents of propylmagnesium chloride in an analogous manner afforded a complex mixture instead of the desired 1,4-addition product.
