# Supplementary material for: The effect of host structure on the selectivity and mechanism of supramolecular catalysis of Prins cyclizations
Source: Chem Sci. 2014 Nov 28;6(2):1383–93. doi: 10.1039/c4sc02735c (PMC5811099; doi:10.1039/c4sc02735c)
Supplement: Supplementary file 1 [file SC-006-C4SC02735C-s001.pdf]

Electronic Supplementary Material (ESI) for Chemical Science.
This journal is © The Royal Society of Chemistry 2014

Supporting Information

The Effect of Host Structure on the Selectivity and Mechanism of Supramolecular Catalysis of Prins Cyclizations

William M. Hart-Cooper, Chen Zhao, Rebecca M. Triano, Parastou Yaghoubi, Haxel Lionel Ozores, Kristen N. Burford, F. Dean Toste,* Robert G. Bergman,* and Kenneth N. Raymond*

Department of Chemistry, University of California, Berkeley, CA 94720 and
Chemistry Division, Lawrence Berkeley National Laboratory, Berkeley, CA, 94720, USA

Table of Contents Page

1. Dependence of k_{obs} on substrate concentration (**5c**, host **2**); order in **5c** (S2)
2. Dependence of k_{obs} on host concentration (**5c**, host **1**); order in **1** (S3)
3. Dependence of k_{obs} on D^+ concentration (**5c**, host **1**); order in D^+ (S4)

1. Relationship between observed rate of cyclization (k_{obs}) versus the concentrations of added substrate.

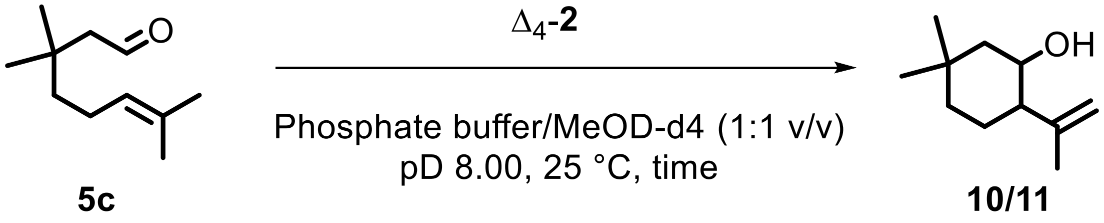

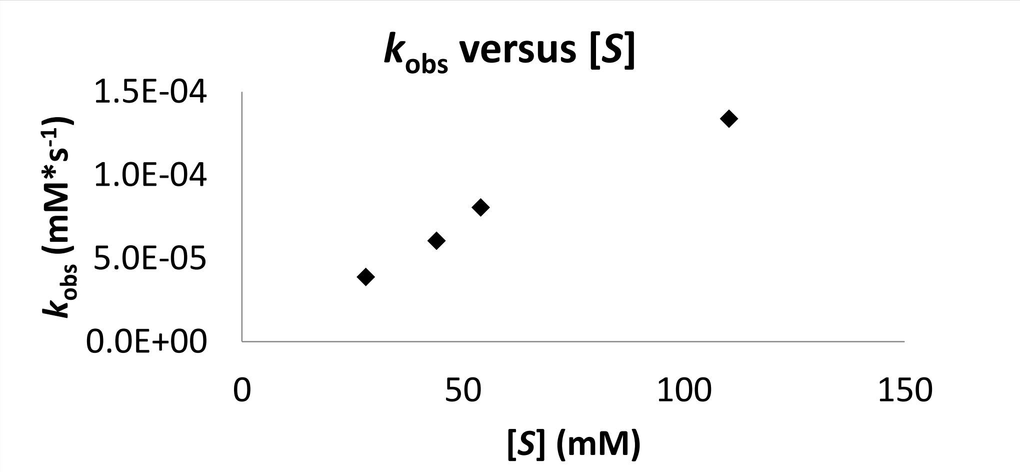

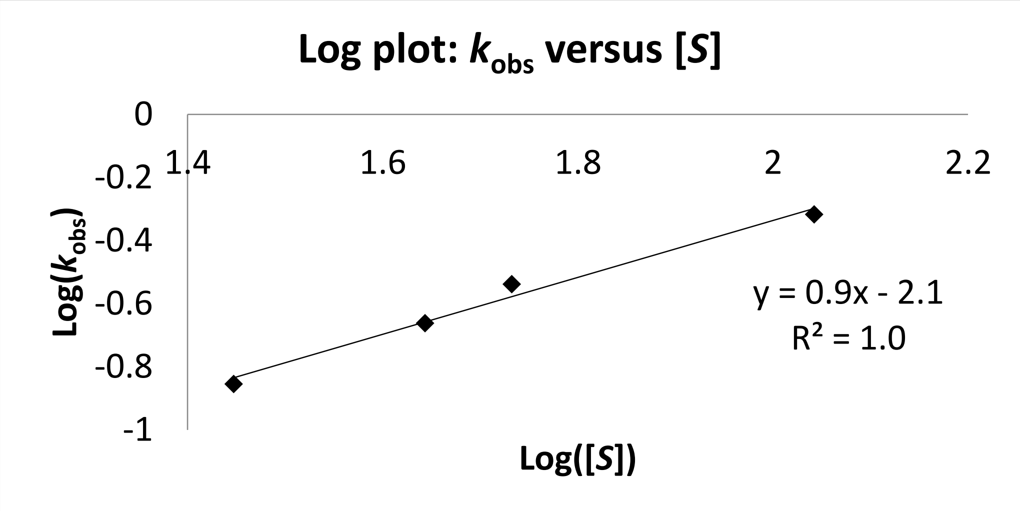

Figure S1. Top: relationship between k_{obs} on substrate concentration ([*S*]). Below: log plot of the same data.

2. Relationship between observed rate of cyclization (k_{obs}) versus the concentrations of added substrate.

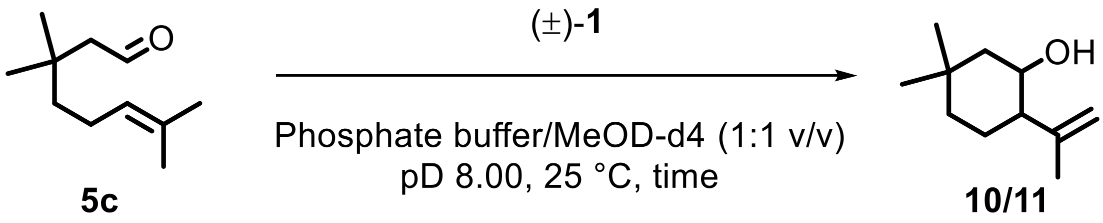

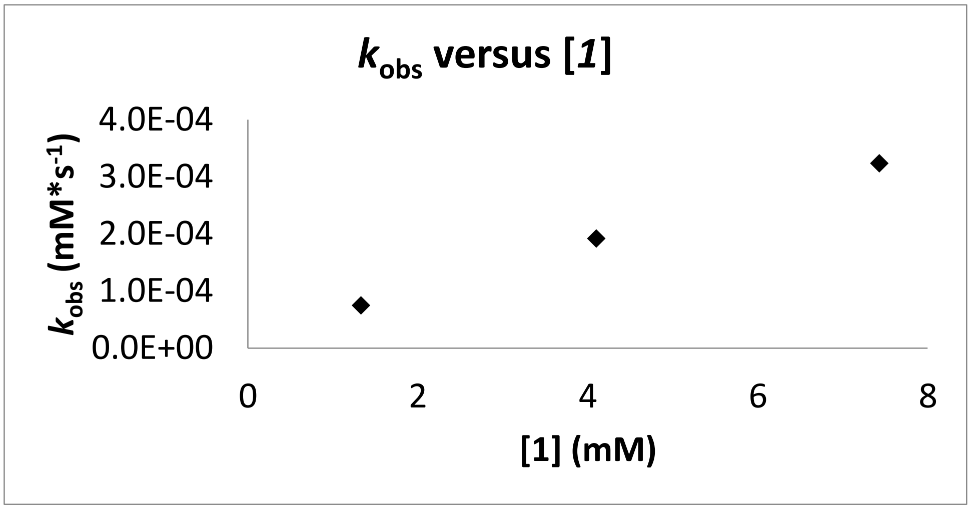

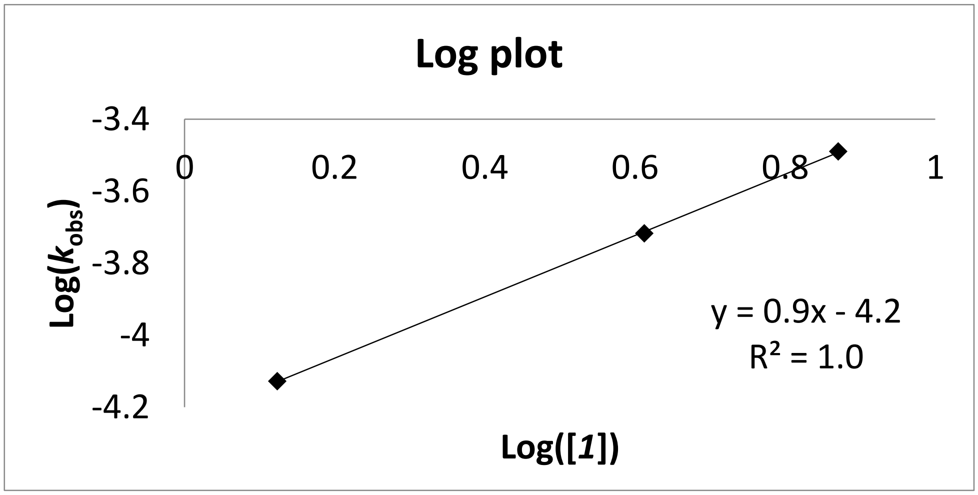

Figure S2. Top: relationship between k_{obs} on host concentration ([*1*]). Below: log plot of the same data.

3. Relationship between observed rate of cyclization (k_{obs}) versus the concentrations of added substrate.

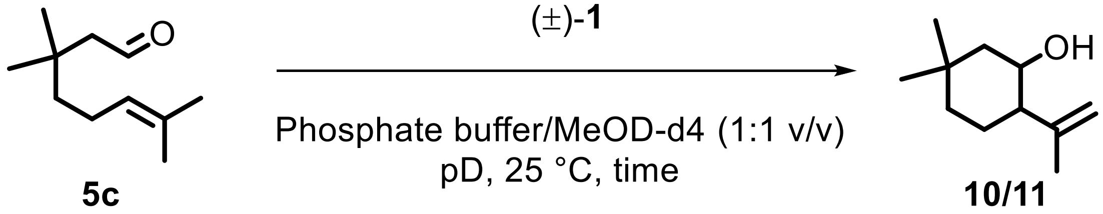

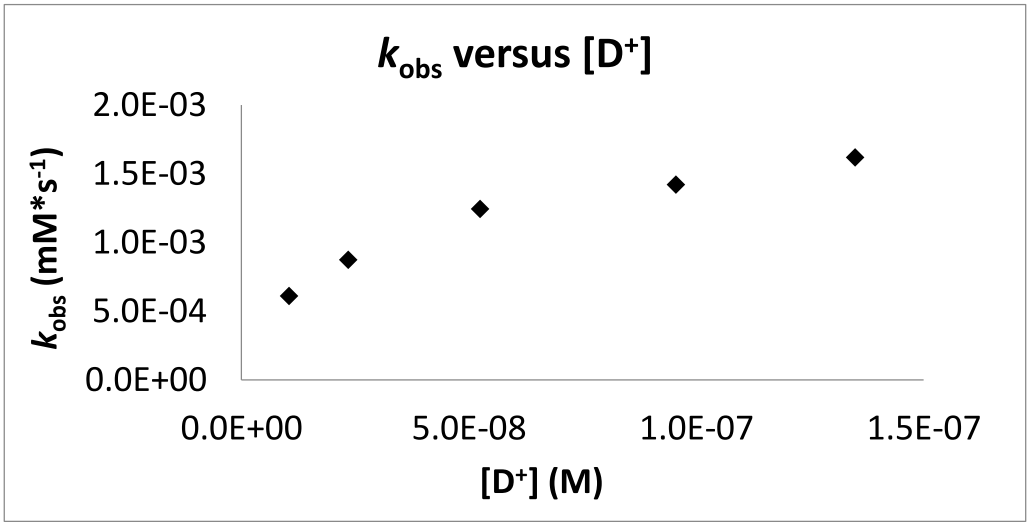

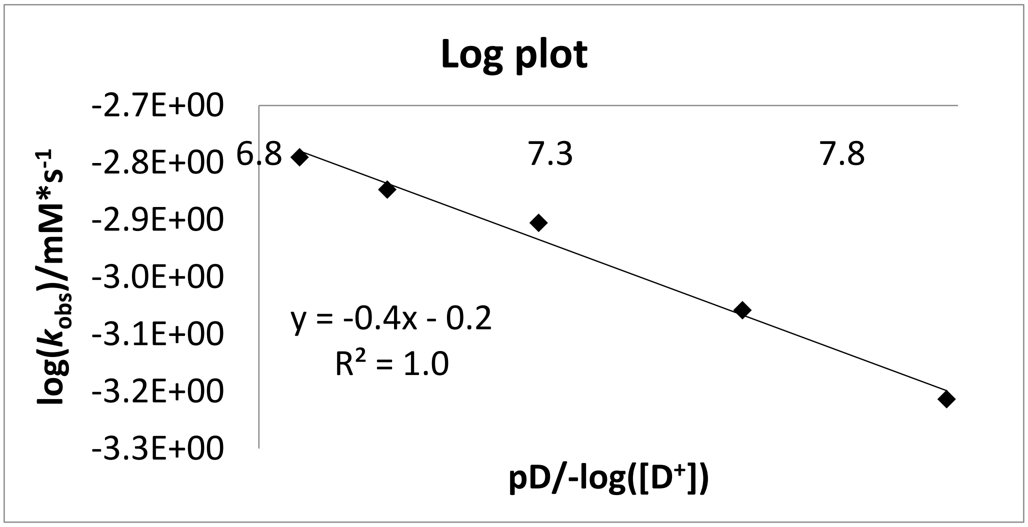

Figure S3. Top: relationship between k_{obs} on bulk solution acidity ([D$^+$]). Below: log plot of the same data.
